# Correlations between Head Ultrasounds Performed at Term-Equivalent Age in Premature Neonates and General Movements Neurologic Examination Patterns

**DOI:** 10.3390/life14010046

**Published:** 2023-12-27

**Authors:** Adrian Ioan Toma, Vlad Dima, Adelina Alexe, Lidia Rusu, Alexandra Floriana Nemeș, Bogdan Florin Gonț, Alexandra Arghirescu, Andreea Necula, Alina Fieraru, Roxana Stoiciu

**Affiliations:** 1 Life Memorial Hospital, 010719 Bucharest, Romaniagontbogdan@gmail.com (B.F.G.);; 2 Faculty of Medicine, University Titu Maiorescu, 040441 Bucharest, Romania; 3 Filantropia Clinical Hospital, Neonatology Department, 011132 Bucharest, Romania; 4 Independent Researcher, 10067 Ploiesti, Romania; 5 Regional Center of Public Health, 700465 Iasi, Romania

**Keywords:** general movements, cramped-synchronized, poor repertoire, premature infants, ultrasound correlations, term-equivalent age, ventricular midbody, basal ganglia width, sinocortical width, anteroposterior diameter of the pons

## Abstract

Background and aim: Our research aims to find correlations between the brain imaging performed at term-corrected age and the atypical general movement (GM) patterns noticed during the same visit a—cramped-synchronized (CS) or poor repertoire (PR)—in formerly premature neonates to provide evidence for the structures involved in the modulation of GM patterns that could be injured and result in the appearance of these patterns and further deficits. Materials and methods: A total of 44 preterm neonates ((mean GA, 33.59 weeks (+2.43 weeks)) were examined in the follow-up program at Life Memorial Hospital Bucharest at term-equivalent age (TEA). The GM and ultrasound examinations were performed by trained and certified specialists. Three GM pattens were noted (normal, PR, or CS), and the measurements of the following cerebral structures were conducted via head ultrasounds: ventricular index, the short and long axes of the lateral ventricles, the midbody distance of the lateral ventricle, the diagonal of the caudate nucleus, the width of the basal ganglia, the width of the interhemispheric fissure, the sinocortical width, the length and thickness of the callosal body, the anteroposterior diameter of the pons, the diameter of the vermis, and the transverse diameters of the cerebellum and vermis. The ultrasound measurements were compared between the groups in order to find statistically significant correlations by using the F_ANOVA_ test (significance *p* < 0.05). Results: The presence of the CS movement pattern was significantly associated with an increased ventricular index (mean 11.36 vs. 8.90; *p* = 0.032), increased midbody distance of the lateral ventricle–CS versus PR (8.31 vs. 3.73; *p* = 0.001); CS versus normal (8.31 vs. 3.34; *p* = 0.001), increased long and short axes of the lateral ventricles (*p* < 0.001), and decreased width of the basal ganglia–CS versus PR (11.07 vs. 15.69; *p* = 0.001); CS versus normal pattern (11.07 vs. 15.15; *p* = 0.0010). The PR movement pattern was significantly associated with an increased value of the sinocortical width when compared to the CS pattern (*p* < 0.001) and a decreased anteroposterior diameter of the pons when compared to both the CS (12.06 vs. 16.83; *p* = 0.001) and normal (12.06 vs. 16.78; *p* = 0.001) patterns. The same correlations were present when the subgroup of infants with a GA ≤ 32 weeks was analyzed. Conclusions: Our study demonstrated that there are correlations between atypical GM patterns (cramped-synchronized—CS and poor repertoire—PR) and abnormalities in the dimensions of the structures measured via ultrasound at the term-equivalent age. The correlations could provide information about the structures that are affected and could lead to a lack of modulation in the GM patterns.

## 1. Introduction

General movements (GMs) are spontaneous movements occurring in fetuses and young infants during the first 6 months of life that represent endogenously generated motor activities that are not provoked by external stimuli [1]. They have been observed in fetuses beginning from the 7th week of fetal life [2,3,4]. To fulfil the established criteria, they should involve the whole body in a variable sequence of arm, neck, leg, and trunk movements, they should wax and wane in intensity, force, and speed, and they should have a variable beginning and end [2]. The normal GM should be fluent and elegant and create an impression of complexity and variability [2].

The origin of GMs is a central pattern generator (CPG) [5] of subcortical origin (brainstem and/or spinal cord) [1,2], modulated by supratruncal structures [2,3,4]. The value in investigating GMs is given by their variability, which is caused by a modulation of CPG activity by cortical and subcortical superior structures [2,3,4]. Indeed, in anencephalic fetuses, GMs are observed, but they lack any variability, which makes them pathologic [6]. The lack of variability and the simultaneous contraction and relaxation of the legs, trunk, and arms is characteristic of an atypical form of GM (cramp-synchronized movements, CS) [1,2] that is strongly associated with a risk of cerebral palsy [7]. This could be explained by the lack of modulation of the CPG by cortical and subplate influences due to lesions [2,3,4]. This movement pattern could be present during the prematurity period (premature infants from 32–34 weeks gestational age (GA) until 1–2 months post-term) [1,2]. Another type of abnormal GM present in premature infants and during the first 1–2 months of life is represented by a poor repertoire (PR) pattern in which the sequence of successive movements is monotonous and lacks complexity and variability [2]. Normal GM patterns at term are called *writhing movements*, and, according to their definition, consist of movements of small to moderate amplitude and slow to moderate speed; their writhing name comes from their elliptical form [2], and this variability is probably the result of modulation of the CPG by superior cortical and subcortical structures [3,4].

The variability represents the main difference between the normal and abnormal general movements (GMs) seen at term [1,2]. The anatomical substrate of abnormal GMs is represented by lesions of the abovementioned modulating structures. There has been research in identifying the structures responsible for modulations in GM patterns [4,8], and the structures involved in the modulation of the GMs noted at term are suggested to be subplate neurons [3,4], a neuronal population that represents an intermediary station in the migration of neurons from the ventricular/subventricular zone to the cortex. This structure begins to regress around term and almost disappears at 6–8 weeks post-term [9].

Our research aims to find correlations between brain imaging performed at term–equivalent age and the atypical GM patterns noticed at that moment (CS or PR) in formerly premature neonates to further provide evidence for the structures involved in the modulation of GM patterns that could be injured, resulting in the appearance of these patterns and further deficits.

The imaging method we chose to investigate the formerly premature neonates at term-equivalent age was head ultrasound. Although former studies have shown that MRI at term is superior to head ultrasound in identifying cerebral lesions in formerly premature neonates [10,11], it is worth mentioning that these studies compared MRI at term with head ultrasound performed at 6 weeks of age. Studies are now showing that head ultrasound performed at TEA on the same day as an MRI has the same value in identifying the main lesions and establishing a prognosis—a score was developed and tested with good results (correspondence with MRI) in this group [12]. Also, the different structures of the brain could be measured at term [13], and the procedure of measurement is standardized [13,14,15]. There has been a good correspondence between the measurements performed using ultrasound and MRI for most brain structures [13]. In addition, in recent years, there has been a controversy regarding the utility of TEA MRI scans in formerly premature infants and the information this procedure could add, with various pros [16] and cons [17].

## 2. Materials and Methods

The study was performed in the follow-up program for high-risk neonates at Life Memorial Hospital Bucharest. The procedures performed (clinical examinations, GM examination, head ultrasound) were part of the standard examination protocol for a premature infant at term-equivalent age (TEA). The approval of the hospital’s Ethics Committee was received prior to data collection. All the parents of the patients involved in the study provided informed consent for the participation and also a separate informed consent for filming the premature infant for the GM examination.

Regarding the inclusion and exclusion criteria, the patients were part of the follow-up program for high-risk neonates conducted at Life Memorial Hospital. This is a second opinion follow-up clinic, where the colleagues send the most difficult cases for follow-up and early intervention. The inclusion criteria were the following:

- TEA between April and 30 August 2023;
- Gestational age between 30–36 weeks;
- Parents’ agreement for the GM assessment and inclusion in the study.

The patients were both inborn or outborn and referred to the clinic.

The exclusion criteria were the gestational age below 30 weeks or over 37 weeks and if the family disagreed to be included in the GM evaluation program.

### 2.1. The General Movements (GM) Examination

The general movements examination was performed according to the guidelines stated by the authors [3,18]. The baby was undressed, lying on a flat white surface, free of colored items or toys, filmed from the feet. To have a valid recording, the infant should be state 3 or 4 Prechtl [1,3,18], with no fussing or crying—in that case, the assessment was stopped, and the child was reassessed later. The duration of each recording was between 3–5 min and was assessed by a trained evaluator (AA), blinded to the results of the ultrasound, not involved in the performance of ultrasound exams, to avoid bias.

The exam was performed during the visit in the follow-up program at 40 weeks of corrected age/term-equivalent age (TEA).

The GM patterns observed were scored as normal (N), poor repertoire (PR), or cramped-synchronized (CS) based on the Gestalt perception [19], according to the definitions as follows:

Normal pattern—writhing movements—small to moderate amplitude and slow to moderate speed, elliptical in form, with nice rotations of the trunk and head [1,3,18].

Poor repertoire pattern—the sequence of successive movements is monotonous and the movements of the parts of the body do not occur in a complex way [1,2,18].

Cramped-synchronized—rigid movements, almost simultaneous contractions of the muscles of limbs and trunk [1,3,18].

### 2.2. Head Ultrasound Measurements at TEA

The head ultrasound was performed at TEA by a trained specialist (AIT), during the same visit. The ultrasound exams were stored and the measurements were performed after the examination, blinded to the patients’ names.

A Vivid S60 (Manufacturer, General Electric HealthCare, Waukesha, West Milwaukee and Madison, WI, USA) was used to perform the examinations. There were used two probes—a microconvex probe with a frequency of 5–7.5 MHz and a linear probe with a frequency of 7–12 MHz. To maintain the safety of the examination, the mechanical index (MI) and thermal index (TI) were maintained below 1.0 [20,21].

All but two measurements were performed on sections through the anterior fontanelle [13,22].

**Figure 1:**
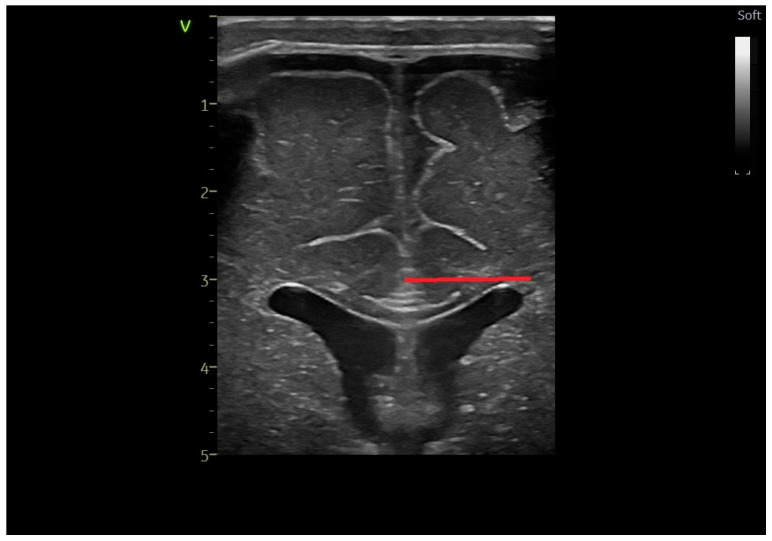
The ventricular index—coronal section at the level of the foramen of Moro, linear probe.

On a coronal view at the level of the foramen of Moro [23], the following structures and diameters were measured:

The ventricular index (Figure 1) is defined as the length of a horizontal line between the midline and the outer border of the lateral ventricle [13,14].

**Figure 2:**
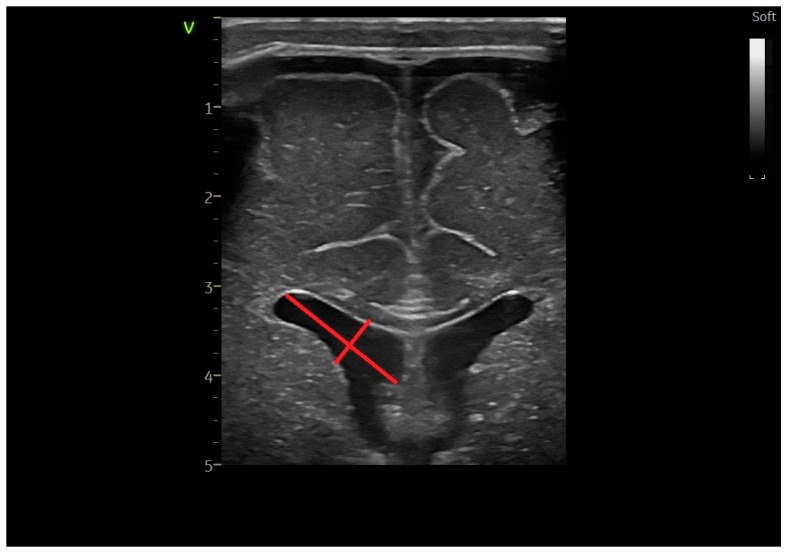
The long and short axes of the frontal horn of the lateral ventricle—coronal section at the level of the foramen of Moro, linear probe.

The long and short axis of the lateral ventricles—frontal horn (Figure 2) is defined as the maximum perpendicular diagonals of the frontal horn [14,24].

**Figure 3:**
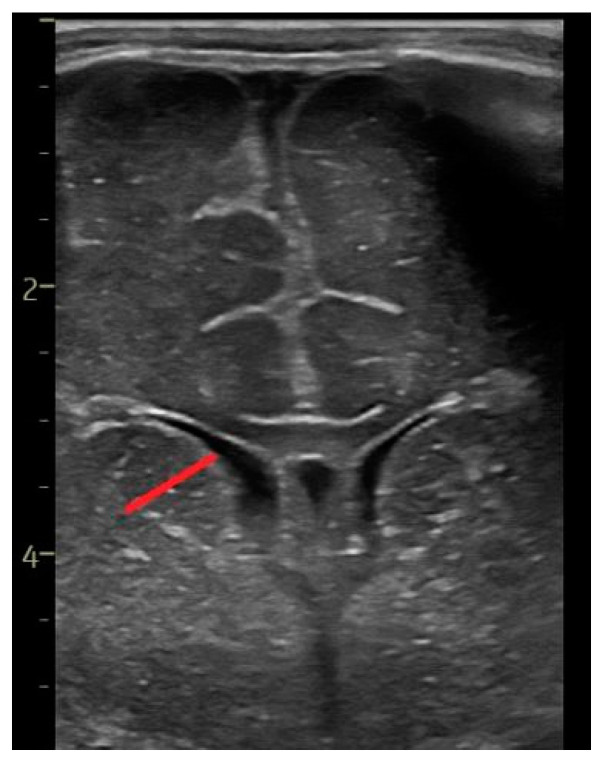
The diagonal of the head of the caudate nucleus—coronal section at the level of the foramen of Moro, linear probe.

The diagonal of the head of the caudate nucleus (Figure 3)—the maximum width perpendicular to the longest diagonal of the head of the caudate, in the coronal section [14,15].

**Figure 4:**
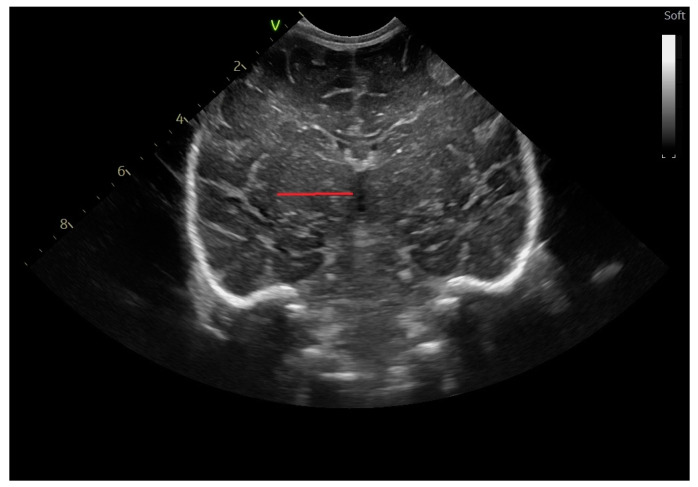
Width of the basal ganglia coronal section at the level of the foramen of Moro, linear probe.

Width of the basal ganglia (Figure 4): the maximum distance between the most lateral border of the basal ganglia and the midline [14,15].

**Figure 5:**
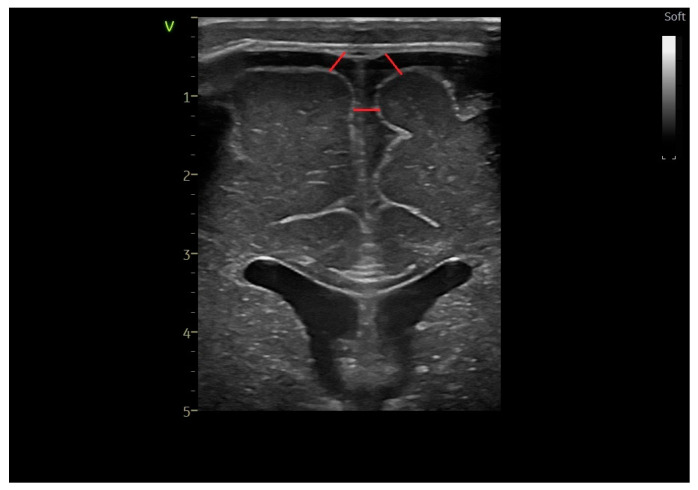
Interhemispheric fissure and sinocortical width—coronal section at the level of the foramen of Moro, linear probe.

Sinocortical width (Figure 5): the distance between the cortex and the superior sagittal sinus.

Width of the interhemispheric fissure (Figure 5): the maximum distance between the hemispheres [14].

**Figure 6:**
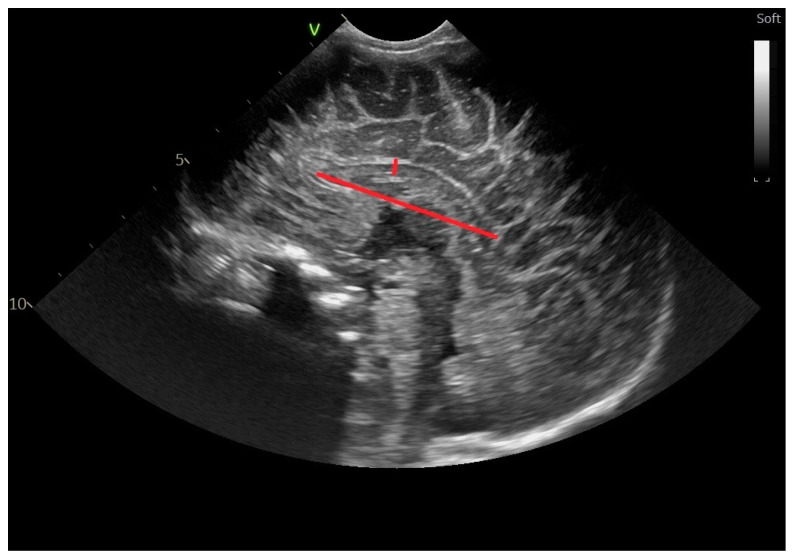
Width and length of the corpus callosum, sagittal section through the anterior fontanelle—microconvex probe.

On a sagittal view, through the anterior fontanelle [23], the following were measured:

Length and thickness of the corpus callosum (Figure 6)—length defined as the distance between the genu and the splenium of the corpus callosum [14,15], thickness measured at the midpart of the corpus callosum [22].

**Figure 7:**
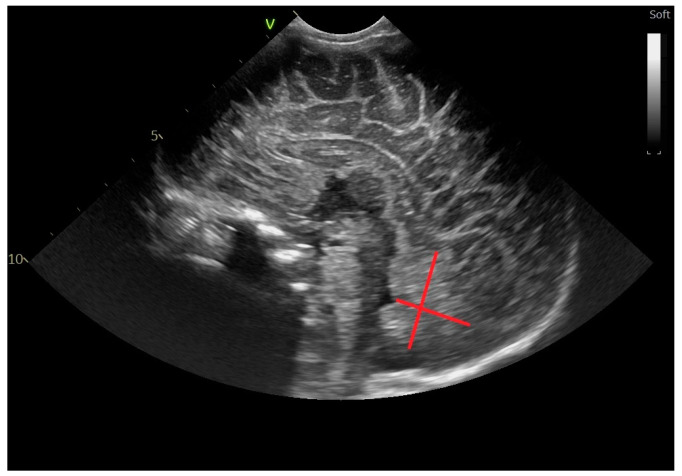
Perpendicular diameters of the cerebellar vermis- sagittal section through the anterior fontanelle; microconvex probe.

Diameters of the cerebellar vermis (Figure 7)—vermis height is defined as the distance between the superior and the inferior borders of the vermis [14,15], and anteroposterior diameter is defined as the diameter perpendicular to the height from the indentation of the IVth ventricle to the most posterior part of the vermis [22].

**Figure 8:**
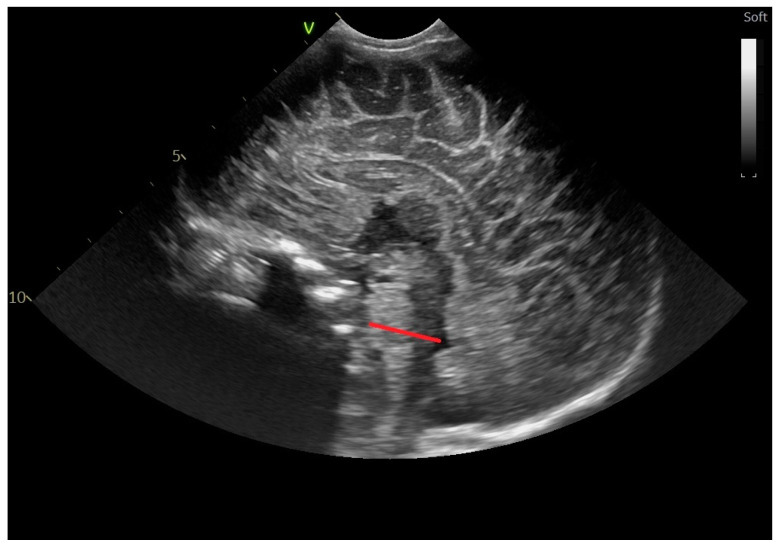
Anteroposterior diameter of the pons-sagittal section through the anterior fontanelle; microconvex probe.

Anteroposterior diameter of the pons (Figure 8)—the distance between the anterior border of the pons and the upper border of the IVth ventricle [14,15].

**Figure 9:**
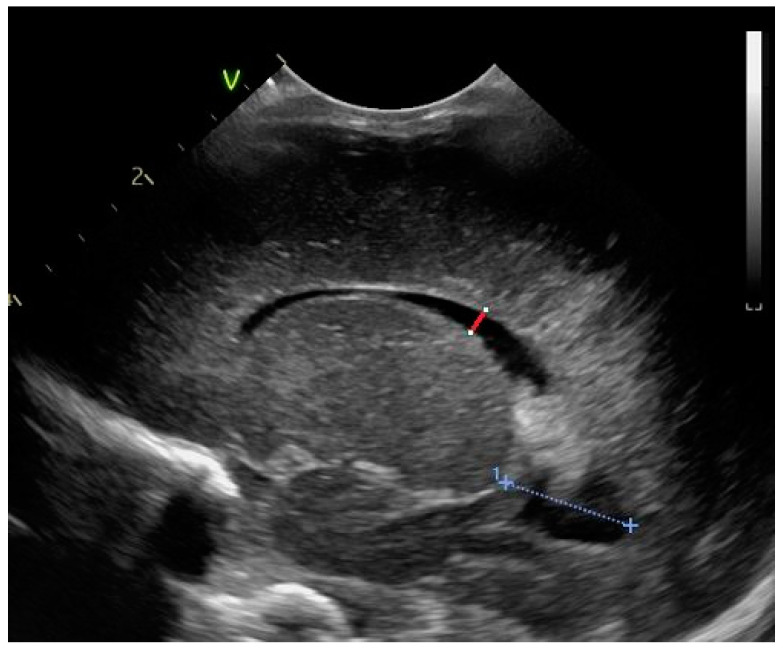
Midbody of the lateral ventricle—parasagittal section through the anterior fontanelle—microconvex probe: red line. The blue line represents the thalamo–occipital distance.

On a parasagittal section through the anterior fontanelle [23], the following were measured: 

The midbody of the lateral ventricle (Figure 9) is defined as the distance between the walls of the inferior and the superior wall of the body of the lateral ventricle, at the level where the ventricle enlarges to form the body of the VL and where the motor pathways project [13,15,22].

**Figure 10:**
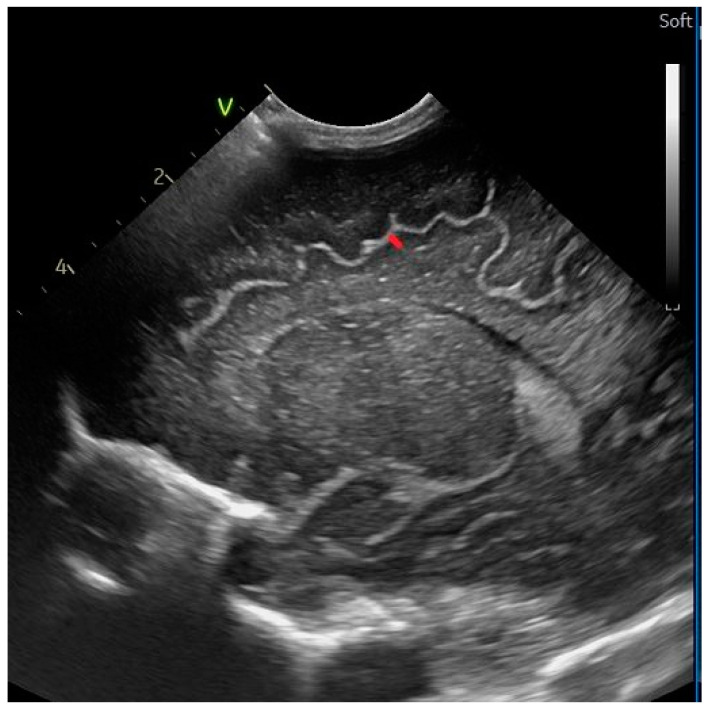
Cortical depth at the level of the cingulate sulcus (red line), parasagittal section through the anterior fontanelle—microconvex probe.

Cortical depth at the level of the cingulate sulcus (Figure 10): the distance measured from the upper hyperechogenic margin to the lower margin is defined as increased echogenicity from the subcortical white matter [13,14].

**Figure 11:**
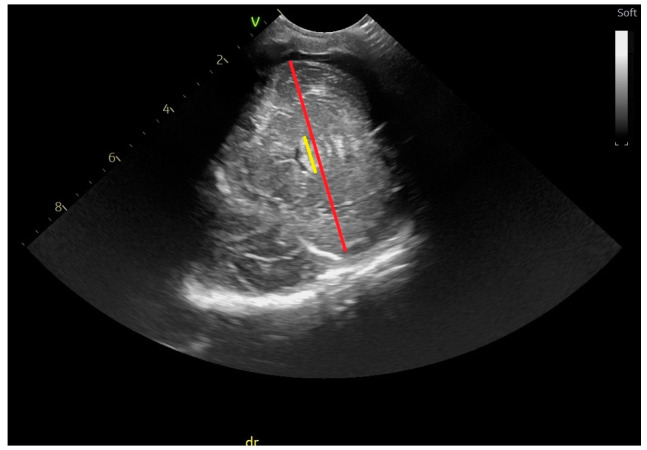
Transverse cerebellar diameter (red) and the diameter of the vermis (yellow)—transverse section, mastoid fontanelle.

The transverse cerebellar diameter was obtained by the mastoid fontanelle window: the maximum diameter was measured via coronal section at the level of the pons; also, at the same level, the transverse diameter of the vermis was measured (Figure 11) [25].

### 2.3. Statistical Analysis of the Data

The data from the patients were collected in a centralized anonymized database that can be accessed upon request at adrian.toma@prof.utm.ro. The statistical analysis of the data was realized in another center by a specialized statistician (LR). SPSS 18.0 was used for this purpose. The statistical significance for the test was chosen to be 95% CI (*p* < 0.05) [26].

The analysis of the correlation between the numeric variables and the GMs patterns was performed using the ANOVA test. The distribution of the variables was checked to confirm a normal distribution. The F_ANOVA_ test was applied to calculate the statistically significant differences between the groups according to the distribution of the series of values in the case of a 95% confidence interval for the quantitative continuous variables. The F_ANOVA_ test was used in the case of three or more groups with a normal distribution, corroborated with the post hoc Turkey HS correction to reduce the errors occurring when more than one hypothesis was tested [26].

The results are represented as a synthetic table.

## 3. Results

### 3.1. Characteristics of the Study Population

A number of 44 premature infants, part of the follow-up program at Life Memorial Hospital, were included in the study. The mean gestational age was 33.59 weeks (±2.43 weeks) and the median was 34 weeks.

**Figure 12:**
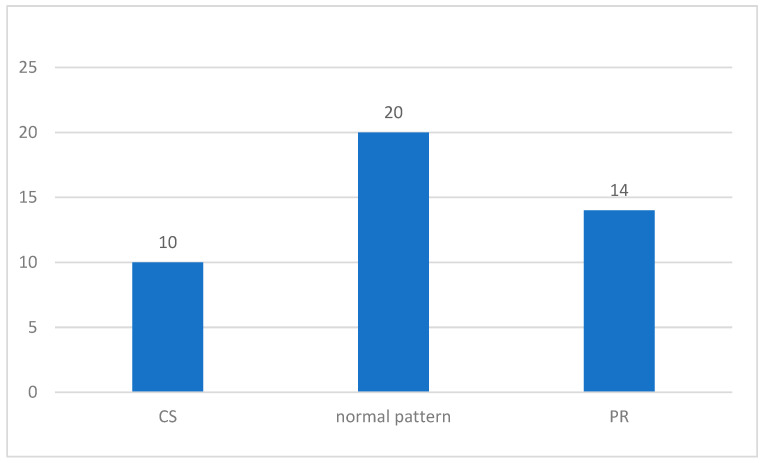
Frequency of General Movements patterns: whole group.

The frequencies of the GMs patterns are shown in Figure 12.

After stratification on gestational age groups, we noted the following (Table 1).

The baseline characteristics of the studied population can be found in Table 2.

It is noticeable that the patients in the CS GMs pattern group have significantly lower gestational age and birth weight than the other two groups, spent more days on parenteral nutrition, and had a longer time of antibiotic treatment. Regarding the PR group, they had lower birth weight than the normal pattern group, but did not differ in other characteristics.

The characteristics of the group regarding the pathologies encountered can be found in Table 3.

It was observed that the only significant difference between the groups was a more frequent occurrence of NEC in the CS and PR group compared with the normal pattern group. The respiratory pathology was increased at the limits of statistical significance (*p* < 0.066). The groups did not differ significantly regarding birth asphyxia, ROP, GM-IVH, or PVL, although for PVL the number of cases diagnosed (3) was too small to make statistical assumptions.

The basic characteristics and the pathology encountered were analyzed separately for the subgroup of patients with gestational ages < 32 weeks (Table 4 and Table 5). No statistically significant differences were noted, except a higher incidence of CPAP in the normal pattern group compared to the CS GMs pattern group, which was at the limit of statistical significance (*p* = 0.05).

### 3.2. Analysis of the Correlations in the Whole Group

After confirming the normal distribution for the analyzed variables, the correlations were checked by using the F_ANOVA_ test (Table 6). The test was first applied to the whole population of patients.

The following statistically significant results were found for the CS movement pattern group:

The ventricular index was significantly higher in the CS pattern group than in the normal pattern group (mean 11.36 vs. 8.90; *p* = 0.032).

The midbody of the lateral ventricle had a higher mean level in the CS group than the PR group (8.31 vs. 3.73; *p* = 0.001) and normal pattern group (8.31 vs. 3.34; *p* = 0.001); the means of the last-mentioned groups were comparable (3.34 vs. 3.73; *p* = 0.889).

The long axis of the lateral ventricle had a higher mean value in the CS group compared with the PR group (15.45 vs. 10.18; *p* = 0.001) and the normal pattern group (15.45 vs. 10.08; *p* = 0.001)—the means of those two groups were, again, comparable.

The short axis of the lateral ventricle had a higher mean value in the CS group compared with the PR group (7.38 vs. 3.22; *p* = 0.001) and the normal pattern group (7.38 vs. 3.61; *p* = 0.001)—the means of those two groups were, again, comparable.

The width of the basal ganglia had a lower mean value in the CS sample compared with the PR group (11.07 vs. 15.69; *p* = 0.001) and the normal pattern group (11.07 vs. 15.15; *p* = 0.0010).

For the PR movement pattern group, the following differences were found to be statistically significant:

The sinocortical width had a higher mean value in the PR movement pattern group compared with both CS (5.53 vs. 2.34; *p* = 0.001) and normal pattern (2.34 vs. 1.90; *p* = 0.454)—there were no differences between the means of the last two groups.

The AP (anteroposterior diameter of the pons) had a lower mean value in the PR group compared with the CS (12.06 vs. 16.83; *p* = 0.001) and normal pattern (12.06 vs. 16.78; *p* = 0.001) groups.

### 3.3. Analysis of the Correlation in the Subgroup of Infants with Gestational Age ≤ 32 Weeks

According to the literature, the risk for the appearance of neurological sequelae is greater at lower gestational ages. [27] We analyzed the correlation between GMs patterns and the findings on head ultrasound performed at TEA in the group of patients with GA ≤ 32 weeks. The results can be visualized in Table 7.

In the case of the CS GMs pattern, we found the same statistically significant correlations with the ventricular index, the midbody of the lateral ventricles, the short and long axes of the lateral ventricles, and the basal ganglia. In addition, the AP diameter of the vermis had the highest mean in the CS group, compared with both the PR group (7.28 vs. 2.88; *p* = 0.001) and the normal pattern group (24.55 vs. 20.38; *p* = 0.04).

Regarding the PR GMs pattern, the same statistically significant differences were noted in the case of the sinocortical width and AP diameter of the pons (Table 5).

## 4. Discussion

We consider the strong points of the study to be the examination of the patients at TEA and the study design that is trying to avoid biases. The simultaneous examination of the patients clinically (GM) and by ultrasound offered the opportunity to detect the lesions that are reflected in clinical abnormalities. As previously mentioned in the introduction, the ultrasound exam at TEA provides data comparable with the MRI at TEA [12,22]. The GM examination and the ultrasound were performed by different people. The ultrasound measurements were accomplished by a person who was not aware of the GMs examinations results, to avoid the detection bias [28]. Also, the statistical analysis of the data was performed by a person from a different center, a statistician without formal medical training. This person received the database and the research questions in order to avoid detection and reporting biases [28].

We were also aware of certain weak points in our research. The first one was the fact that this was a monocentric study. Our future aim is, though, to involve more centers in a study that will validate our findings The small number of patients involved represents another weak point that could be corrected in a future multicenter validation study. Also, another weak point is the lack of very small premature infants in our research. We had in our group more than one-third of the infants with gestational ages less than or equal to 32 weeks, and the results do not differ essentially between the whole group and the subset of small premature neonates. We could have followed the premature infants to the age of fidgety movements (3–5 months) [1,2,3,18]. Indeed, after finding the results of this research, we began to enroll another cohort to examine them at both TEA and 3–5 months to see if the ultrasound correlates with the presence or absence of the fidgety movements. In the next cohort, the MOS (Motor Optimality Score) [29] and GMOS (General Movements Optimality Score) [30] scores will be established.

Regarding the MRI at 40 weeks corrected age, this exam was not performed. This could also represent a weak point of our research. There were, however, several considerations regarding this option. As specified in a policy statement of the American Academy of Pediatrics [17], the issue of the 40 weeks corrected age MRI is controversial and is not recommended by this board. Although the opinion of the Newborn Brain Society is that the MRI should be performed at 40 weeks corrected age [16], we decided not to perform this. As mentioned in the text of the paper, there is a good correlation between the TEA MRI and ultrasound performed at the same age [12,22].

As expected, the baseline characteristics of the groups and the incidence of different pathologies were somehow different between CS, PR, and normal GM patterns. This has also been demonstrated by earlier studies [1,2,3]. It is to be remembered, though, that the aim of the study was not to identify these differences, but to find if abnormal GM patterns were also associated with certain abnormal values in the head ultrasound performed at TEA.

The CS movement pattern has been significantly associated with two kinds of anomalies: signs of ventricular dilatation, probably reflecting a white matter lesion [12,22], and a reduced diameter of the basal ganglia. We could speculate that these findings bring us data about the origin of the modulation of the GM patterns and the mechanism by which this movement pattern occurs in premature neonates that will subsequently develop motor deficits/cerebral palsy. We should remember that the migration of the neurons occurs during the second half of the pregnancy and the first migration station is represented by the subplate neurons [8]. These subplate neuron structures have a maximum of development between 28 and 34 weeks of gestation and then they are regressing [31]. It seems that the CPG-generated activity in the subcortical structures is modulated by this subplate and this modulation gives the variability of the normal writhing movements [3,4,8]. The majority of severe neurologic injuries in preterm neonates occurs in the periventricular zone and affects both periventricular white matter and the subplate neurons [32,33], resulting in white matter disorder that is defined as white matter lesion and the associated neuronal and axonal deficits [33]. As a consequence, the structures that modulate the GMs are destroyed, and an unmodulated pattern of GM results. The increase of the ventricular index, frontal horn axes, and midbody distance are markers of white matter lesions in premature infants and are, except for the ventricular index, a part of the scoring system for quantifying the white matter injury at TEA, with a good correlation with MRI [12,22]. In conclusion, the increased size of the ventricles associated with a CS movement pattern reflects a white matter lesion that destroys the subplate neurons and, in this way, results in an un-modulated GM pattern.

The finding of a decreased dimension of the basal ganglia is more difficult to explain. There have been studies showing an association between the decreased dimensions of the basal ganglia and thalami in the term neonate with birth asphyxia and the occurrence of CS GM pattern [7,34]. In this subset of patients, the affected structures that modulate the CPG could be the basal ganglia, diminished in dimension because fewer synapses occur there due to neuronal and white matter damage.

Even if the results of our study allow the abovementioned speculations, it is our belief that further and more in-depth studies are needed to support the hypothesis of an involvement of less-developed structures in the genesis of GMs and the role of their destruction in the atypical GM patterns observed in certain patients.

The PR movement pattern is special because there is not a direct link between its presence and neuromotor consequences—it can evolve to CS, normal, fidgety, or no fidgety movements [1,2,3]. Accordingly, the findings in this subgroup are more difficult to interpret. The statistically significant differences noted between the PR group and the other two groups were a larger sinocortical width and a shorter AP diameter of the pons. For the first variable (the sinocortical width), we could speculate that the larger value reflects a degree of nonoptimal development of the gray or/and white matter, leading to a monotonous set of movements that could lead to PR movement patterns; there is a known correlation between an enlarged subarachnoid space in the premature infant examined at TEA and the risk of developmental delay [35]. For the AP diameter of the pons, we could not find such a correlation in the literature. However, we could speculate that either the subcortical CPG or the modulating pathway are affected, leading to a limited repertoire of movements.

It is important to note that the statistically significant correlations between GM patterns and the results of ultrasound at TEA are also present when we analyze the group at highest risk, the premature infants with GA less or equal to 32 weeks [27].

The explanation for the high incidence of the patients with abnormal GM patterns in our study group is that the center is a second-opinion follow-up clinic, where the colleagues send the most difficult cases for follow-up and early intervention. We focused on babies with higher gestational ages because many of the studies undertaken now are focused on micropreemies (less than 28 weeks gestational age), and the category of premature infants with GA greater or equal to 30 weeks seems to be studied less and the number of such premature infants is much larger than the others, so the data obtained have a greater applicability in the general population of premature infants.

The two patients with GA greater than 32 weeks deserve a separate discussion:

The patient with a gestational age of 33 weeks was a male SGA neonate (birth weight of 1100 g), born via caesarean section with perinatal asphyxia (APGAR 7) in which a prenatal grade II Volpe germinal matrix–intraventricular hemorrhage was identified at birth (prenatal origin). He was ventilated for 48 h. The baby also presented with sepsis (*Klebsiella pneumoniae*) treated with a 10-day course of antibiotics.

The second patient was the second of twins, female, with an apparent initial normal course in the maternity that presented on day 10 of life with bloody stools and NEC that was treated conservatively and did not need surgery. The evolution was slowly favorable.

The premature infants included in the study were examined between April and August 2023. As a consequence, we cannot tell for the moment which one will develop cerebral palsy. They are still patients in the follow-up program and their status at 2 years corrected age will be also published, though this is not the primary end-point of our study. We are currently enrolling premature infants in another cohort in which the endpoint will be neurological status at 2 years corrected age.

In addition to the abovementioned, there is a need for a continuation of follow-up of these patients until the ages when fidgety movements can be observed and the age when cerebral palsy can be diagnosed. As previously mentioned, it is necessary to confirm these findings by a multicentric replication study and by research involving infants born at lower gestational ages.

## 5. Conclusions

Our study demonstrates that there are correlations between the atypical GM patterns (cramped-synchronized—CS and poor repertoire—PR) and abnormalities in the dimensions of the structures measured by ultrasound at term-equivalent age. The cramp-synchronized movement pattern was associated with increased dimensions of the lateral ventricles (ventricular index, diagonals of the frontal horn, and midbody distance) that are known to be markers of the lesions of the white matter and with a decreased diameter of the basal ganglia. The PR movement pattern was associated with an increased sinocortical width and with a decreased AP diameter of the pons. The correlations could give information about the structures that are affected and could lead to the lack of modulation in the GM patterns (subplate neurons, basal ganglia in the case of CS pattern and cortical structures in the case of the PR pattern).

## Figures and Tables

**Table 1:** Distribution of GMs patterns among gestational age (GA) groups.

GA Group	CS	PR	Normal
≤32 weeks	8	6	3
33–34 week	2	3	3
35–36 weeks	0	5	14
Total	10	14	20

Legend: GA—gestational age; GMs—general movements; CS—cramped-synchronized; PR—poor repertoire.

**Table 2:** Basic characteristics of the groups according to GMs patterns—whole group.

Parameters	Pattern			*p*-Values for F_ANOVA_ Test
	Normal (*n* = 20)	CS * (*n* = 10)	PR ** (*n* = 14)	
Gestational age—weeks x ± SD, median/limits	34.80 ± 1.64 35/31–37	31.90 ± 1.20 ^b)^ 32/30–34	33.07 ± 3.15 ^d) d)^ 33/26–36	0.003
Birth weight—grams x ± SD, median/limits	2302 ± 729 2303/1400–4100	1199 ± 283 ^a)^ 1199/900–1900	1842 ± 702 ^d) c)^ 1841/1100–3300	0.001
Parenteral nutrition—days x ± SD, median/limits	2.75 ± 2.83 3/0–10	7.00 ± 3.30 ^c)^ 7/1–12	5.71 ± 5.74 ^d) d)^ 6/0–15	0.020
Antibiotic treatment—days x ± SD, median/limits	5.05 ± 5.11 5/0–24	10.80 ± 1.95 ^c)^ 11/5–26	7.93 ± 6.46 ^d) d)^ 8/0–21	0.050
CPAP—hours x ± SD, median/limits	23.40 ± 6.48 23/0–120	16.80 ± 8.04 ^d)^ 17/0–72	14.14 ± 6.09 ^d) d)^ 5.75/3.70–7.00	0.581
Mechanical Ventilation—days x ± SD, median/limits	2.94 ± 1.19 3/0–4.8	1.60 ± 0.70 ^d)^ 1.60/0–5	1.46 ± 0.69 ^d) d)^ 1.45/0–7	0.843

Post-hoc Tukey HSD; * CS vs. normal pattern. ** PR vs. normal pattern and PR vs. CS. a) *p* < 0.001. b) *p* < 0.01. c) *p* < 0.05. d) *p* > 0.05.

**Table 3:** Characteristics of the group—pathology.

Associated Diseases	Pattern			Chi-Square Test Likelihood Ratio
	Normal (*n* = 20)	CS * (*n* = 10)	PR ** (*n* = 14)	
Necrotizing enterocolitis (NEC)	0 (0.0%)	2 (20.0%) ^c)^	3 (21.4%) ^c) d)^	0.037
Asphyxia	1 (5.0%)	2 (20.0%) ^d)^	2 (14.3%) ^d) d)^	0.422
Retinopathy of prematurity	2 (10.0%)	3 (30.0%) ^d)^	2 (14.3%) ^d) d)^	0.396
Head ultrasound results No hemorrhage or PVL Germinal matrix/intraventricular hemorrhage PVL LSV	14 (70.0%) 4 (20.0%) 0 (0.0%) 2 (10.0%)	5 (50.0%) ^d)^ 2 (20.0%) ^d)^ 1 (10.0%) ^d)^ 2 (20.0%) ^d)^	7 (50.0%) ^d) d)^ 5 (35.7%) ^d) d)^ 2 (14.3%) ^d) d)^ 0 (0.0%) ^d) d)^	0.180
Respiratory distress				0.066
No respiratory distress	4 (20.0%)	0 (0.0%) ^d)^	4 (28.6%) ^d) d)^	
RDS	5 (25.0%)	5 (50.0%) ^d)^	7 (50.0%) ^d) d)^	
TTN	11 (55.0%)	5 (50.0%) ^d)^	3 (21.4%) ^d) d)^	

Chi2 test. * CS vs. normal pattern. ** PR vs. normal pattern and PR vs. CS. a) *p* < 0.001. b) *p* < 0.01. c) *p* < 0.05. d) *p* > 0.05. Legend: PVL—periventricular leukomalacia. LSV—lenticulostriate vasculopathy. RDS—respiratory distress syndrome. TTN—transient tachypnea of the newborn.

**Table 4:** Basic characteristics of the groups according to GMs patterns—patients ≤ 32 weeks.

Parameters	Pattern			*p*-Values for F_ANOVA_ Test
	Normal (*n* = 3)	CS * (*n* = 8)	PR ** (*n* = 6)	
Gestational age—weeks x ± SD, median/limits	31.67 ± 0.58 31.50/31–32	31.50 ± 0.93 ^d)^ 31/30–32	30.17 ± 2.56 ^d) d)^ 30/26–32	0.299
Birth weight—grams x ± SD, median/limits	1500 ± 132 1500/1400–1650	1116 ± 158 ^d)^ 1116/900–1350	1617 ± 838 ^d) d)^ 1616/1100–3300	0.210
Parenteral nutrition—days x ± SD, median/limits	4.67 ± 2.52 5/2–7	7.63 ± 3.16 ^d)^ 7.50/1–12	9.17 ± 5.95 ^d) d)^ 9/0–15	0.362
Antibiotic treatment—days x ± SD, median/limits	9.33 ± 2.08 9/7–11	11.50 ± 6.61 ^d)^ 11.50/7–26	13.17 ± 5.19 ^d) d)^ 13/7–21	0.636
CPAP—hours x ± SD, median/limits	52.00 ± 34.18 52/12–120	15.00 ± 9.00 ^c)^ 15/0–72	12.00 ± 8.20 ^b) d)^ 5.75/3.70–7.00	0.050
Mechanical ventilation—days x ± SD, median/limits	2.0 ± 2.0 2–4	2.0 ± 0.82 ^d)^ 1.60/0–5	3.40 ± 1.44 ^b) d)^ 3.30/0–7	0.125

Post hoc Tukey HSD. * CS vs. normal pattern. ** PR vs. normal pattern and PR vs. CS. a) *p* < 0.001. b) *p* < 0.01. c) *p* < 0.05. d) *p* > 0.05.

**Table 5:** Characteristics of the group—pathology of patients ≤ 32 weeks.

Associated Diseases	Pattern			Chi-Square Test Likelihood Ratio
	Normal (*n* = 3)	CS * (*n* = 8)	PR ** (*n* = 6)	
Necrotizing enterocolitis (NEC)	0 (0.0%)	2 (33.3%) ^d)^	1 (12.5%) ^d) d)^	0.337
Asphyxia	1 (33.3%)	2 (25.0%) ^d)^	1(16.7%) ^d) d)^	0.849
Retinopathy of prematurity	1 (33.3%)	3 (37.5%) ^d)^	2 (33.3%) ^d) d)^	0.984
Head ultrasound results No hemorrhage or PVL Germinal matrix/intraventricular hemorrhage PVL LSV	1 (33.3%) 1 (33.3%) 0 (0.0%) 1 (33.3%)	4 (50.0%) ^d)^ 2 (25.0%) ^d)^ 1 (12.5%) ^d)^ 1 (12.5%) ^d)^	2 (33.3%) ^d) d)^ 3 (50.0%) ^d) d)^ 1 (16.7%) ^d) d)^ 0 (0.0%) ^d) d)^	0.690
Respiratory distress				0.127
No respiratory distress	0 (0.0%)	0 (0.0%) ^d)^	1 (16.7%) ^d) d)^	
RDS	2 (66.7%)	4 (50.0%) ^d)^	8 (83.3%) ^d) d)^	
TTN	1 (33.3%)	4 (50.0%) ^d)^	0 (0.0%) ^d) d)^	

Chi2 test. * CS vs. normal pattern. ** PR vs. normal pattern and PR vs. CS. a) *p* < 0.001. b) *p* < 0.01. c) *p* < 0.05. d) *p* > 0.05. Legend: PVL—periventricular leukomalacia. LSV—lenticulostriate vasculopathy. RDS—respiratory distress syndrome. TTN—transient tachypnea of the newborn.

**Table 6:** Analysis of the correlation between GMs patterns and the measurements of structures by head ultrasound at term-equivalent age—whole group.

Parameters	Pattern			*p*-Values for F_ANOVA_ Test
	Normal (*n* = 20)	CS * (*n* = 10)	PR ** (*n* = 14)	
Ventricular measurements				
Ventricular index x ± SD, median/limits	8.90 ± 2.57 9.90/1.30–12.10	11.36 ± 2.10 ^c)^ 11.05/9.0–14.80	9.44 ± 2.43 ^d) d)^ 9.55/6.10–15.40	0.039
Midbody VL x ± SD, median/limits	3.34 ± 2.44 3.25/0.10–10.50	8.31 ± 3.20 ^a)^ 7.45/5.60–16.10	3.73 ± 1.58 ^d) a)^ 3.45/1.50–6.60	0.001
Frontal horn—long axis x ± SD, median/limits	10.08 ± 2.88 9.85/5.10–14.60	15.45 ± 2.11 ^a)^ 15.65/12.0–19.50	10.18 ± 2.80 ^d) a)^ 9.70/6.40–14.70	0.001
Frontal horn—short axis x ± SD, median/limits	3.61 ± 1.21 3.70/1.0–5.30	7.38 ± 1.70 ^a)^ 7.50/3.80–9.90	3.22 ± 1.27 ^d) a)^ 3.10/1.50–5.40	0.001
Subarachnoid space				
Sino-cortical width x ± SD, median/limits	1.90 ± 0.97 1.45/0.60–4.00	2.34 ± 0.95 ^d)^ 2.40/1.20–4.10	5.53 ± 0.91 ^a) a)^ 5.75/3.70–7.00	0.001
Inter-hemispheric fissure x ± SD, median/limits	1.56 ± 0.88 1.35/0.30–3.70	2.12 ± 1.40 ^d)^ 1.40/0.80–4.40	2.37 ± 1.29 ^d) d)^ 2.35/0.40–5.00	0.123
Parenchima				
Cortical thickness x ± SD, median/limits	1.52 ± 0.39 1.50/0.70–2.20	1.81 ± 0.31 ^d)^ 1.75/1.50–2.50	1.55 ± 0.44 ^d) d)^ 1.50/1.0–2.20	0.143
Subcortical structures				
Width of the basal ganglia x ± SD, median/limits	15.15 ± 1.95 15.05/10.20–18.60	11.07 ± 0.94 ^a)^ 11.05/9.50–12.30	15.69 ± 1.87 ^d) a)^ 16.05/11.10–17.90	0.001
Diagonal of the head of the caudate nucleus x ± SD, median/limits	5.77 ± 0.98 5.40/4.40–8.10	5.60 ± 0.87 ^d)^ 5.65/4.20–6.90	5.54 ± 0.83 ^d) d)^ 5.45/4.0–7.40	0.755
Corpus callosum				
Corpus callosum thickness—body x ± SD, median/limits	2.29 ± 0.69 2.20/1.0–3.70	2.46 ± 0.50 ^d)^ 2.40/1.60–3.00	2.41 ± 0.48 ^d) d)^ 2.35/1.50–3.10	0.715
Corpus callosum length x ± SD, median/limits	45.21 ± 3.87 45.55/38.30–51.30	44.99 ± 3.53 ^d)^ 45.70/39.20–49.80	43.16 ± 4.70 ^d) d)^ 43.50/33.80–51.00	0.333
Posterior fossa				
AP diameter of the pons x ± SD, median/limits	16.78 ± 2.11 16.70/12.50–20.30	16.83 ± 1.42 ^d)^ 16.75/14.90–19.60	12.06 ± 1.71 ^a) a)^ 11.90/9.0–15.50	0.001
Vermis height x ± SD, median/limits	28.33 ± 4.39 29.0/13.60–33.80	31.44 ± 3.63 ^d)^ 32.30/24.90–36.50	29.97 ± 3.37 ^d) d)^ 30.40/22.90–34.90	0.123
Vermis AP diameter x ± SD, median/limits	23.04 ± 3.48 22.05/18.70–33.10	23.96 ± 3.23 ^d)^ 23.55/18.40–27.80	22.11 ± 2.43 ^d) d)^ 21.90/18.0–27.20	0.363
Cerebellar transverse diameter x ± SD, median/limits	58.40 ± 5.53 58.50/49.60–67.10	59.31 ± 7.74 ^d)^ 59.50/48.90–68.40	57.24 ± 7.36 ^d) d)^ 57.00/46.80–68.90	0.748
Vermis diameter x ± SD, median/limits	13.67 ± 1.71 13.20/11.20–17.50	13.00 ± 1.32 ^d)^ 13.00/10.60–15.20	12.61 ± 1.65 ^d) d)^ 12.60/9.00–14.50	0.174

Post hoc Tukey HSD. * CS vs. normal pattern. ** PR vs. normal pattern and PR vs. CS a) *p* < 0.001. b) *p* < 0.01. c) *p* < 0.05. d) *p* > 0.05 Legend: CS—cramped-synchronized; PR—poor repertoire; AP—anteroposterior; VL—lateral ventricle.

**Table 7:** Analysis of the correlation between GMs patterns and the measurements of structures by head ultrasound at term-equivalent age—infants with gestational age ≤ 32 weeks.

Parameter	Group			*p*-Values for F_ANOVA_ Test
	Normal (*n* = 3)	CS * (*n* = 8)	PR ** (*n* = 6)	
Ventricular measurements				
Ventricular index x ± SD, median/limits	9.47 ± 2.57 9.40/6.50–11.0	11.40 ± 2.37 ^d)^ 11.40/9.0–14.80	8.52 ± 1.77 ^d) d)^ 8.50/26.0–32.0	0.080
Midbody VL x ± SD, median/limits	3.43 ± 1.44 3.45/1.80–4.50	8.53 ± 3.59 ^c)^ 8.50/5.60–16.10	3.58 ± 1.42 ^d) c)^ 3.45/1.60–6.0	0.007
Frontal horn—long axis x ± SD, median/limits	9.73 ± 2.45 10.0/7.30–12.20	15.27 ± 2.34 ^c)^ 15.10/12.0–19.50	10.32 ± 2.94 ^d) b)^ 10.10/7.40–14.70	0.004
Frontal horn –short axis x ± SD, median/limits	4.80 ± 0.50 4.90/4.30–5.30	7.28 ± 1.91 ^d)^ 7.20/3.80–9.90	2.88 ± 1.38 ^d) a)^ 3.0/1.50–5.20	0.001
Subarachnoid space				
Sino-cortical width x ± SD, median/limits	2.83 ± 1.39 3.00/1.30–4.00	2.60 ± 0.88 ^d)^ 2.40/1.20–4.10	5.13 ± 0.74 ^b) a)^ 5.14/4.10–5.90	0.001
Inter-hemispheric fissure x ± SD, median/limits	2.07 ± 1.42 2.00/1.20–3.70	2.30 ± 1.52 ^d)^ 2.20/0.80–4.40	2.18 ± 1.63 ^d) d)^ 2.10/0.40–5.00	0.973
Parenchima				
Cortical thickness x ± SD, median/limits	1.67 ± 0.42 1.50/1.20–2.00	1.83 ± 0.35 ^d)^ 1.85/1.50–2.50	1.58 ± 0.50 ^d) d)^ 1.00/1.0–2.10	0.530
Subcortical structures				
Width of the basal ganglia x ± SD, median/limits	14.40 ± 0.53 14.50/14.0–15.0	11.10 ± 1.06 ^c)^ 11.05/9.50–12.30	14.90 ± 2.63 ^d) b)^ 15.0/11.10–17.60	0.003
Diagonal of the head of the caudate nucleus x ± SD, median/limits	5.73 ± 1.02 6.10/5.0–6.90	5.89 ± 0.70 ^d)^ 5.69/4.80–6.90	5.85 ± 0.90 ^d) d)^ 5.85/4.70–7.40	0.963
Corpus callosum				
Corpus callosum thickness—body x ± SD, median/limits	2.47 ± 1.08 2.50/1.70–3.70	2.56 ± 0.46 ^d)^ 2.50/2.0–3.0	2.18 ± 0.47 ^d) d)^ 2.38/1.50–2.90	0.504
Corpus callosum length x ± SD, median/limits	41.10 ± 3.48 41.0/38.30–45.0	44.93 ± 3.85 ^d)^ 45.0/39.20–49.80	40.90 ± 6.06 ^d) d)^ 41.0/33.80–50.00	0.259
Midbody VL x ± SD, median/limits	3.43 ± 1.44 3.45/1.80–4.50	8.53 ± 3.59 ^c)^ 8.50/5.60–16.10	3.58 ± 1.42 ^d) c)^ 3.45/1.60–6.0	0.007
Posterior fossa				
AP diameter of the pons x ± SD, median/limits	14.73 ± 1.93 15.0/12.50–15.90	17.15 ± 1.37 ^d)^ 17.0/15.20–19.60	12.13 ± 1.31 ^c) a)^ 12.0/10.30–13.80	0.001
Vermis height x ± SD, median/limits	28.70 ± 1.73 29.0/27.60–30.70	32.53 ± 2.98 ^d)^ 32.50/26.40–36.50	30.57 ± 3.98 ^d) d)^ 30.40/24.90–34.90	0.224
Vermis AP diameter x ± SD, median/limits	22.20 ± 2.69 22.15/20.0–25.20	24.55 ± 3.38 ^d)^ 24.0/18.40–27.80	20.38 ± 1.83 ^d) c)^ 20.45/18.0–23.0	0.048

Post hoc Tukey HSD. * CS vs. normal pattern. ** PR vs. normal pattern and PR vs. CS a) *p* < 0.001. b) *p* < 0.01. c) *p* < 0.05. d) *p* > 0.05 Legend: CS—cramped-synchronized; PR—poor repertoire; AP—anteroposterior; VL—lateral ventricle.

## Data Availability

The database of the study can be accessed upon request at the address adrian.toma@prof.utm.ro.
